# Reference gene selection for RT-qPCR analysis in *Harmonia axyridis*, a global invasive lady beetle

**DOI:** 10.1038/s41598-018-20612-w

**Published:** 2018-02-09

**Authors:** Xiaowei Yang, Huipeng Pan, Ling Yuan, Xuguo Zhou

**Affiliations:** 1 Department of Entomology, University of Kentucky, Lexington, KY 40546 USA; 2 Department of Entomology, Cornell University, Geneva, NY 14456 USA; 3 Department of Entomology, South China Agricultural University, Key Laboratory of Bio-Pesticide Innovation and Application of Guangdong Province, Guangzhou, China; 4 Department of Plant and Soil Sciences, University of Kentucky, Lexington, KY 40546 USA

## Abstract

*Harmonia axyridis* is a voracious predator, a biological control agent, and one of the world most invasive insect species. The advent of next-generation sequencing platforms has propelled entomological research into the genomics and post-genomics era. Real-time quantitative PCR (RT-qPCR), a primary tool for gene expression analysis, is a core technique governs the genomic research. The selection of internal reference genes, however, can significantly impact the interpretation of RT-qPCR results. The overall goal of this study is to identify the reference genes in the highly invasive *H. axyridis*. Our central hypothesis is that the suitable reference genes for RT-qPCR analysis can be selected from housekeeping genes. To test this hypothesis, the stability of nine housekeeping genes, including 18*S, 28S, ACTB, ATP1A1, GAPDH, HSP70, HSP90, RP49*, and *ATP6V1A*, were investigated under both biotic (developmental time, tissue and sex), and abiotic (temperature, photoperiod, *in vivo* RNAi) conditions. Gene expression profiles were analyzed by *geNorm, Normfinder, BestKeeper*, and the ΔCt method. Our combined results recommend a specific set of reference genes for each experimental condition. With the recent influx of genomic information for *H. axyridis*, this study lays the foundation for an in-depth omics dissection of biological invasion in this emerging model.

## Introduction

The multicolored Asian lady beetle, *Harmonia axyridis* (Coleoptera: Coccinellidae), a generalist predator, preys on aphids and scale insects on crops and other plants^1^. *Harmonia axyridis* is native to central and eastern Asian. To exploit its ecosystem services, numerous releases were attempted in North America and Europe, as early as 1916^2,3^. Due to its broad range of preys and incredible consumption rate, *H. axyridis* indeed has been used to control aphids^4–6^ and other sap-sucking arthropod pests^7,8^. However, the worldwide propagation of *H. axyridis* threatens the indigenous lady beetles and other non-target species^9–11^. Considered as “the most invasive ladybird on Earth”, the role of *H. axyridis* has shifted from a global biological control agent to an invasive alien species^12^. Multiple factors contribute to this transition. Predation of eggs and larvae of other lady beetle species is one of the reasons which leads to the decline of native species^13,14^. A higher level of resistance to infection is the other major reason to benefit its competition in the field^15–17^. The molecular basis of this resistance, however, is poorly understood.

Double-stranded RNA (dsRNA) can induce sequence-specific posttranscriptional gene silencing in many organisms, i.e., RNA interference (RNAi)^18,19^. RNAi can not only investigate gene functions *in vivo* or *in vitro*, but also offers a novel approach with a brand new mode-of-action to control arthropod pests^20–24^. With a recent influx of genomic information for *H. axyridis*, there is an increasing need for the development of genetic tools to functionally interpret the sequencing data^20,24–26^.

Real-time quantitative PCR (RT-qPCR) has been used primarily for gene expression quantification^27–29^. RT-qPCR analysis is highly sensitive, and its accuracy can be affected by RNA quantity, transcription efficiency, amplification efficiency and experimental procedures between samples. To avoid biases, normalization of gene expression is an essential step^30^. The most common practice is to compare a target gene expression with an internal reference gene^31^. Housekeeping genes, such as *beta-actin* (*ACTB*), *glyceraldehyde-3-phosphate dehydrogenase* (*GAPDH*), and *translation elongation factor 1-alpha* (*EF1A*)^32,33^ have been used extensively for RT-qPCR analysis. However, under any given experimental condition, the expression of these commonly used reference genes may vary substantially^34–37^. A systematic and customized study for each tested species is recommended for identifying appropriate reference genes^38,39^.

The overall goal of this study is to identify candidate reference genes in the highly invasive *H. axyridis*. Our objective is to determine the suitable reference genes for RT-qPCR analysis in *H. axyridis* from selected housekeeping genes, an array of constitutively expressed genes maintaining the basic cellular functions in an organism. We evaluated the stability of nine housekeeping genes under selected biotic and abiotic conditions, respectively. The candidate genes include *18S ribosomal RNA*(*18S*), *28S ribosomal RNA* (*28S*), *Na*+*/K*+-*ATPase subunit alpha 1* (*ATP1A1*), *heat shock protein 70* (*HSP70*), *heat shock protein 90* (*HSP90*), *ribosomal protein 49* (*RP49*), *V-ATPase subunit A* (*ATP6V1A*), *ACTB* and *GAPDH* from *H. axyridis*. All these housekeeping genes have been used empirically as the reference genes for RT-qPCR analyses in other organisms, especially in insects. The specific environmental conditions range from biotic (developmental stage, tissue type, and sex) to abiotic treatments (temperature, photoperiod, and *in vivo* RNAi). As a result, a specific set of reference genes is recommended for each given condition.

## Results

### RT-qPCR analysis

**Figure 1:**
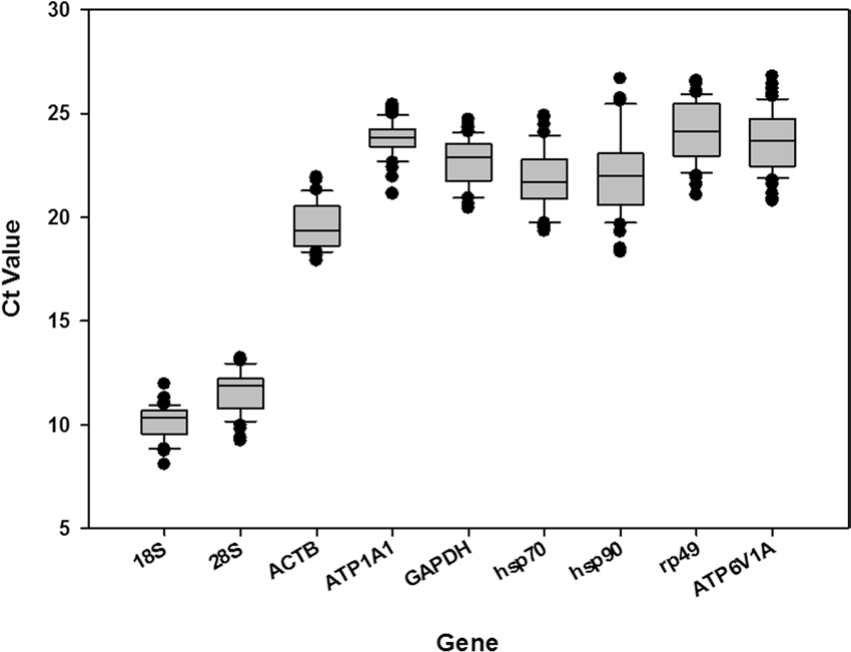
Ct value of candidate reference genes in *H. axyridis*. The Ct values of candidate reference genes in all tested samples were documented. The dot indicates the maximum or minimum value of replicated samples, while whiskers indicate the standard error of the mean.

For each candidate reference gene, a single amplicon was produced, as detected by agarose gel electrophoresis analysis and the melting curve analysis. Nonspecific bands were not found, and a single peak was observed in the melting curve analysis. A standard curve was generated for each gene, using a five-fold serial dilution of the pooled cDNA. Efficiency of RT-qPCR ranged between 90 and 110% (Table 1), which is considered standard^40^. Ct values of the nine candidate reference genes ranged from 8 to 27, covering all the experimental conditions (Fig. 1). While the vast majority of Ct values were found between 17 and 26, *18S* was the most abundant transcript. *ATP1A1*, *VATP6V1A*, and *RP49* were the least abundant candidate reference genes.

**Table 1:** Primer sequence, amplicon length and RT-qPCR analysis of candidate reference genes and a target gene.

Genes	Accession Number	Primer Sequence	Amplicon Length(bp)	PCR Efficiency	Regression Coefficient
*Candidate reference gene*					
*18S*	GU073689.1	AAGACGGACAGAAGCGAAAG	100	1.029	0.9999
		GGTTAGAACTAGGGCGGTATCT			
*28S*	FJ621330.1	ACCCGAAAGATGGTGAACTATG	101	1.025	0.9995
		CCAGTTCCGACGATCGATTT			
*ACTB*	MF785104	ACCCATCTACGAAGGTTATGC	122	1.005	0.9962
		CGGTGGTGGTGAAAGAGTAA			
*ATP1A1*	AY303371.1	CCGTAACTGGTGATGGTGTT	111	1.066	0.981
		GGATCATATCTGCCGCTTGT			
*GAPDH*	MF785103	TGACTACAGTTCACGCAACC	140	1.060	0.9754
		GATGACTTTGGTTACAGCCTTTG			
*HSP70*	EF668009.1	CCAAAGACAGGCTACCAAAGA	101	0.982	0.9989
		TGTCCAAACCGTAGGCAATAG			
*HSP90*	FJ501962.1	CGCCTTCCAAGCAGAAATTG	135	1.078	0.9847
		GTGAGAGACTGGTAACGGATTT			
*RP49*	AB552923.1	GCCGTTTCAAGGGACAGTAT	84	0.972	0.998
		TGAATCCAGTAGGAAGCATGTG			
*ATP6V1A*	MF785105	GAGTTGGGTCCTGGTATTATGG	126	1.093	0.9989
		AGTTCTGGACAAACAAGGTACA			
*Target gene*					
*TPS*	FJ501960.1	CATACTATAATGGTGCGTGTAATG	144	0.943	0.9985
		ATTTAAGGGCTTTGATTGTGC

### Stability of candidate reference genes under biotic conditions

**Figure 2:**
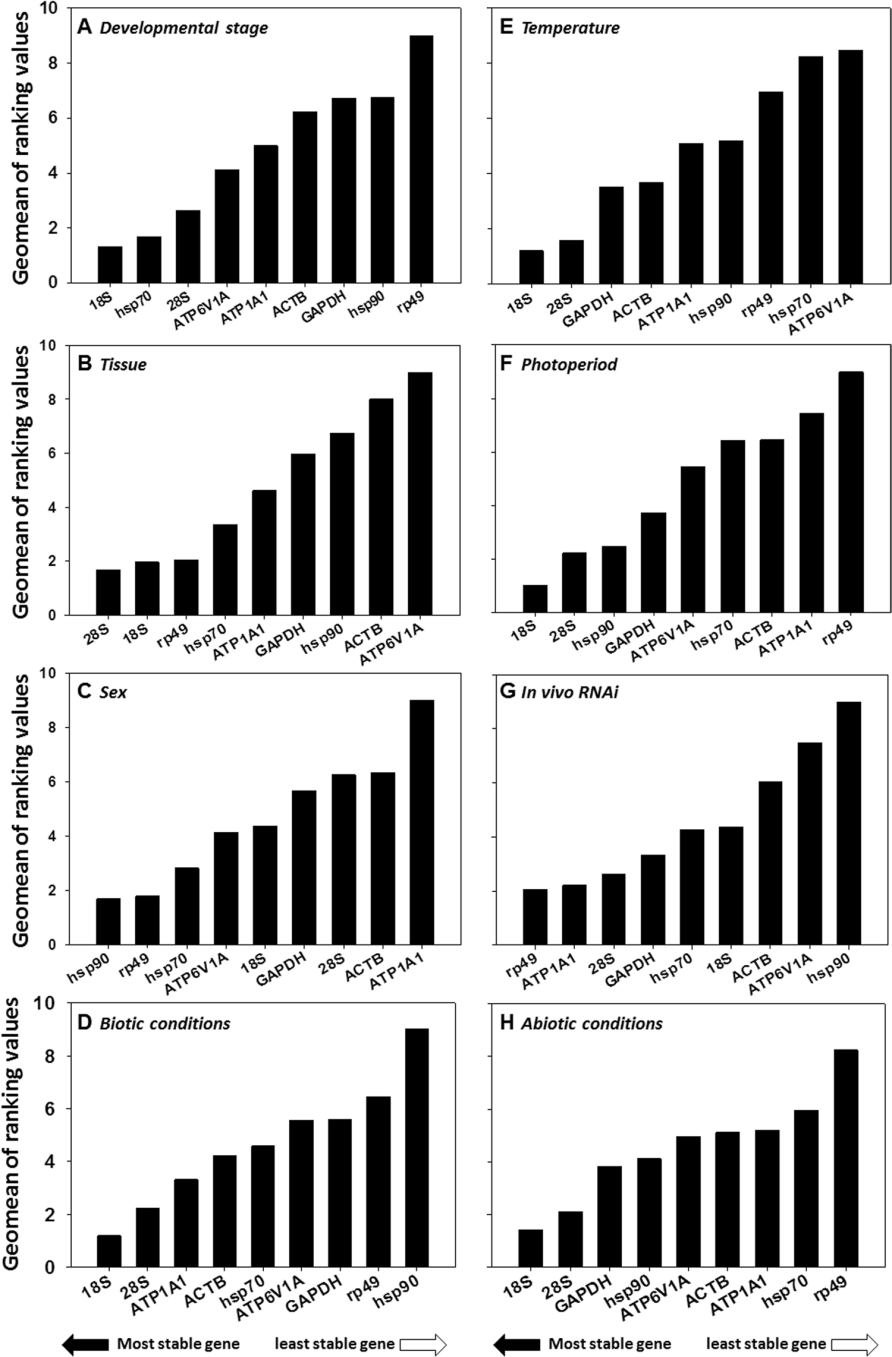
Stability of candidate reference gene expression under biotic and abiotic experimental conditions. (**A**) Development stage, (**B**) Tissue, (**C**) Sex, (**D**) Biotic factors, (**E**) Temperature, (**F**) Photoperiod, (**G**) *In vivo* RNAi, and (**H**) Abiotic factors. A lower Geomean value suggests stable expression.

For different developmental stages, geNorm ranked the stability from high to low as 18S = HSP70, 28S, ATP6V1A, ATP1A1, ACTB, HSP90, GAPDH, and RP49. Normfinder provided a ranking as 18S, HSP70, ATP6V1A, 28S, ATP1A1, ACTB, HSP90, GAPDH, and RP49. Bestkeeper offered a list as follows: 18S, HSP70, 28S, ATP1A1, GAPDH, HSP90, ACTB, ATP6V1A, and RP49 (Table 2). The best set of reference genes was recommended in Table 2. Integrating the results from all four programs, RefFinder identified the consensus top three candidates, 18S, HSP70 and 28S, across different developmental stages. 18S was the most stable gene, while RP49 was the least stable candidate (Table 2, Fig. 2A).

**Table 2:** Stability of candidate reference genes in response to biotic conditions.

Biotic Conditions	Candidate Genes	*geNorm*		*Normfinder*		*BestKeeper*		*ΔCt*		Recommendation
		Stability	Ranking	Stability	Ranking	Stability	Ranking	Stability	Ranking	
Development stage	*18S*	0.674	1	0.596	1	0.54	1	1.2	1	*18S, HSP70, 28S*
	*28S*	0.855	3	0.929	4	0.82	3	1.33	4	
	*ACTB*	1.21	6	1.09	6	1.03	7	1.46	6	
	*ATP1A1*	1.174	5	0.997	5	0.93	4	1.4	5	
	*ATP6V1A*	1.078	4	0.861	3	1.09	8	1.33	3	
	*GAPDH*	1.34	8	1.201	8	0.93	5	1.55	8	
	*HSP70*	0.674	1	0.75	2	0.76	2	1.25	2	
	*HSP90*	1.268	7	1.157	7	0.99	6	1.5	7	
	*RP49*	1.407	9	1.333	9	1.12	9	1.64	9	
Tissue	*18S*	0.138	1	0.455	5	0.14	1	0.73	3	*28S, 18S, RP49*
	*28S*	0.138	1	0.436	4	0.17	2	0.72	1	
	*ACTB*	0.73	8	0.939	8	0.98	8	1.09	8	
	*ATP1A1*	0.579	6	0.381	3	0.45	5	0.77	5	
	*ATP6V1A*	0.871	9	1.272	9	1.2	9	1.36	9	
	*GAPDH*	0.526	5	0.568	6	0.66	7	0.82	6	
	*HSP70*	0.451	4	0.373	2	0.42	4	0.74	4	
	*HSP90*	0.637	7	0.592	7	0.55	6	0.87	7	
	*RP49*	0.351	3	0.356	1	0.36	3	0.73	2	
Sex	*18S*	0.604	5	0.634	6	0.32	2	0.85	6	*HSP90, RP49*
	*28S*	0.673	6	0.821	8	0.46	4	0.95	8	
	*ACTB*	0.746	8	0.601	5	0.69	8	0.82	5	
	*ATP1A1*	0.821	9	0.999	9	1.02	9	1.08	9	
	*ATP6V1A*	0.368	3	0.424	4	0.58	6	0.72	4	
	*GAPDH*	0.706	7	0.768	7	0.35	3	0.93	7	
	*HSP70*	0.197	1	0.372	3	0.6	7	0.7	3	
	*HSP90*	0.453	4	0.231	1	0.18	1	0.67	2	
	*RP49*	0.197	1	0.283	2	0.49	5	0.67	1

For different tissues, the consensus top three candidates were *28S, 18S* and *RP49* according to *RefFinder* (Table 2, Fig. 2B). Specifically, *28S* and *ATP6V1A* were the most and the least stable genes, respectively. For different sexes, the top three most stable candidates in both sexes were *HSP90*, *RP40*, and *HSP70* according to *RefFinder* (Table 2, Fig. 2C). *HSP90* and *ATP1A1* were the most and the least stable genes, respectively. Based on the comprehensive ranking of *RefFinder*, the most to the least stable candidate reference genes under the biotic conditions was: *18S, 28S, ATP1A1, ACTB, HSP70, ATP6V1A, GAPDH, RP49*, and *HSP90* (Table 2; Fig. 2D).

### Stability of candidate reference genes under abiotic conditions

According to *RefFinder*, the consensus top three candidate reference genes under different temperature regime were *18S*, *28S* and *GAPDH* (Table 3, Fig. 2E). Specifically, *18S* and *ATP6V1A* was the most and least stable candidate, respectively. For different photoperiods, the top three candidates were *18S, 28S* and *HSP90* (Table 3, Fig. 2F), in which *18S* and *RP49* was the most and the least stable candidates, respectively. For *in vivo* RNAi experiments, the top three candidates were *RP49*, *ATP1A1*, and *28S* (Table 3, Fig. 2G), in which *RP49* and *HSP90* was the most and the least stable candidates, respectively. Based on the comprehensive ranking of *RefFinder*, the most to the least stable candidate reference genes under the abiotic conditions was: *18S*, *28S*, *GAPDH*, *HSP90*, *ATP6V1A*, *ACTB*, *ATP1A1*, *HSP70*, and *RP49* (Table 3; Fig. 2H).

**Table 3:** Stability of candidate reference genes in response to abiotic conditions.

Biotic Conditions	Candidate Genes	*geNorm*		*Normfinder*		*BestKeeper*		*ΔCt*		Recommendation
		Stability	Ranking	Stability	Ranking	Stability	Ranking	Stability	Ranking	
Temperature	*18S*	0.287	1	0.276	1	0.19	2	0.55	1	*18S, 28S, GAPDH*
	*28S*	0.287	1	0.322	3	0.14	1	0.55	2	
	*ACTB*	0.35	3	0.438	5	0.27	3	0.63	4	
	*ATP1A1*	0.396	4	0.535	7	0.34	4	0.67	6	
	*ATP6V1A*	0.648	9	0.683	9	0.57	7	0.81	9	
	*GAPDH*	0.429	5	0.285	2	0.38	5	0.56	3	
	*HSP70*	0.603	8	0.635	8	0.65	9	0.77	8	
	*HSP90*	0.494	6	0.424	4	0.52	6	0.63	5	
	*RP49*	0.552	7	0.502	6	0.58	8	0.68	7	
Photoperiod	*18S*	0.28	1	0.17	1	0.15	1	0.65	1	*18S, 28S, HSP90*
	*28S*	0.28	1	0.35	4	0.27	2	0.69	3	
	*ACTB*	0.558	6	0.671	6	0.68	8	0.86	6	
	*ATP1A1*	0.695	8	0.714	8	0.55	6	0.94	8	
	*ATP6V1A*	0.521	5	0.592	5	0.62	7	0.8	5	
	*GAPDH*	0.486	4	0.344	3	0.41	4	0.7	4	
	*HSP70*	0.626	7	0.688	7	0.42	5	0.9	7	
	*HSP90*	0.436	3	0.288	2	0.38	3	0.68	2	
	*RP49*	0.841	9	1.257	9	0.93	9	1.35	9	
*In vivo* RNAi	*18S*	0.303	5	0.284	6	0.18	2	0.44	6	*RP49, ATP1A1*
	*28S*	0.283	4	0.246	4	0.11	1	0.41	3	
	*ACTB*	0.365	7	0.537	8	0.2	3	0.59	8	
	*ATP1A1*	0.227	1	0.201	3	0.23	4	0.4	2	
	*ATP6V1A*	0.406	8	0.395	7	0.51	8	0.52	7	
	*GAPDH*	0.227	1	0.271	5	0.35	5	0.44	5	
	*HSP70*	0.325	6	0.175	2	0.38	7	0.42	4	
	*HSP90*	0.489	9	0.744	9	0.79	9	0.78	9	
	*RP49*	0.263	3	0.107	1	0.37	6	0.39	1	

### Recommended reference genes

**Figure 3:**
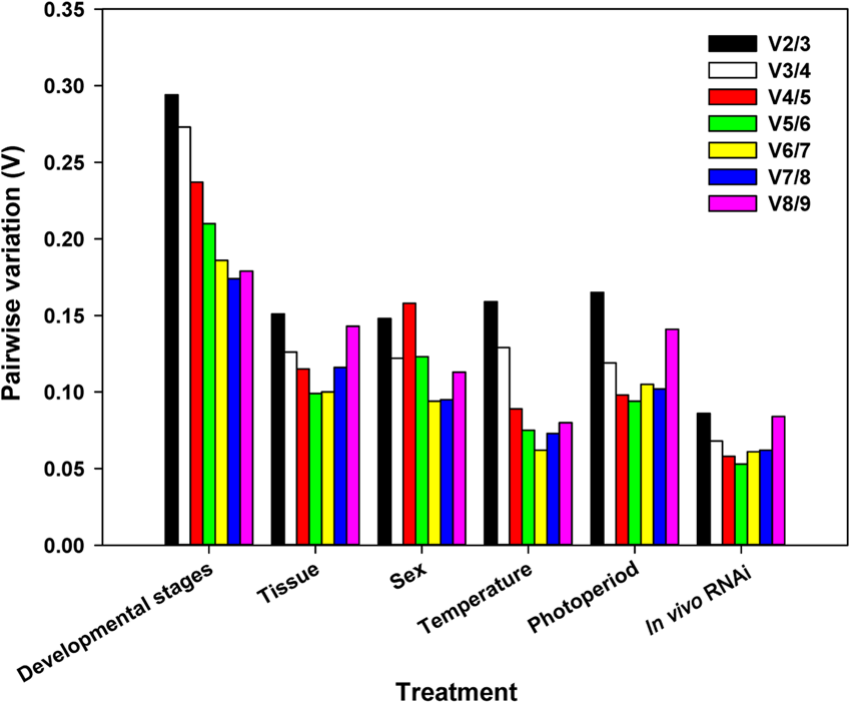
Optimal number of reference genes required for accurate normalization of gene expression. Based on geNorm analysis, average pairwise variations are calculated between the normalization factors NFn and NFn + 1. Values less than 0.15 indicate that n + 1 genes are not required for the normalization of gene expression.

For repeatable and consistent results, multiple normalizers (≥2 reference genes) are required for RT-qPCR analysis. GeNorm analysis evaluated all pairwise variations under each experimental conditions (Fig. 3). According to Vandesompele *et al*.^31^, a Vn/Vn + 1 cutoff value of 0.15 means the addition of n + 1 reference gene is not necessary, i.e., the first n references genes are sufficient to normalize qRT-PCR results. The optimal number of reference genes was recommended in Tables 2 and 3, respectively, for biotic and abiotic conditions. Specifically, for different developmental stages, the recommended reference genes were *18S, HSP70*, and *28S*. For different tissues, the recommendation was *28S, 18S*, and *RP49*. For different sexes, the recommendation was *HSP90* and *RP49*. For different temperature treatments, the recommendation was *18S, 28S*, and *GAPDH*. For different photoperiods, the recommendation was *18S, 28S*, and *HSP90*. Finally, for *in vivo* RNAi, the best combination was *RP49* and *ATP1A1*.

### Validation of selected reference genes

**Figure 4:**
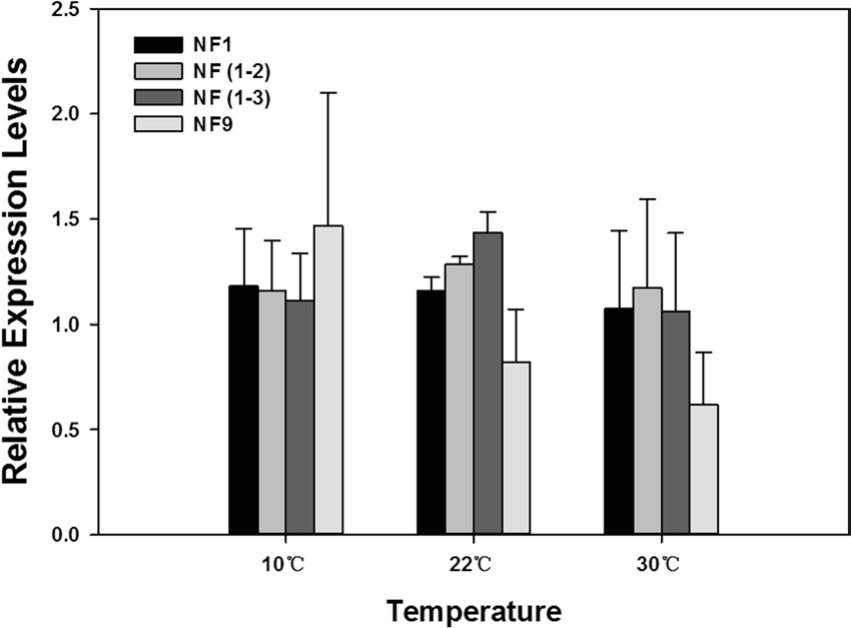
Validation of the recommended reference gene(s). Expression profiles of *TPS* under different temperature treatments were investigated using different normalization factors. Bars represent the means ± standard error of three biological replicates.

The expression of *TPS*, a target gene, was evaluated to validate the recommended reference genes under different temperature treatments. Using the most stable reference gene *18S* (NF 1), the top two stable reference genes *18S* and *28S* (NF 1–2), and the top three stable reference genes, *18S, 28S*, and *GAPDH* (NF 1–3) for normalization, *TPS* expression profiles were similar throughout all three temperature regimes (Fig. 4). In comparison, when *ATP6V1A*, the least stable candidate (NF 9), was used as the reference gene, *TPS* expression patterns were inconsistent across different temperature treatments. Specifically, *TPS* expression was numerically higher at 10 °C, and lower at 22 and 30 °C (Fig. 4).

## Discussion

RT-qPCR has been used extensively for quantification of mRNA expression and is a primary tool for genetic research. Although multiple factors, such as RNA extraction, storage, cDNA synthesis, and handling of materials and reagents, can affect the RT-qPCR analysis, a reliable reference gene (set) to overcome confounding variations in an empirical dataset is of particular importance. Normalization by internal controls is an integral part of the quantification process. A single or multiple stably expressed reference genes are required for the normalization process to achieve accurate and reliable results. Each candidate reference gene should be evaluated under specific experimental conditions to ensure a constant level of expression^35^. Following the “Minimum Information for Publication of Quantitative Real-Time PCR Experiments” (MIQE) guideline^41^, reference gene selection study has been carried out for many insect species^34,42,43^, and has become a routine practice to standardize RT-qPCR analysis.

Due to different algorithms, stability ranking derived from the four analytical tools can vary. For example, when *H. axyridis* was injected with dsRNAs (*in vivo* RNAi), *28S* was rated as the best reference gene by *BestKeeper*, *RP49* was considered as the most stable by *Normfinder* as well as ΔCT method, whereas *ATP1A1* and *GAPDH* were the top choice by *geNorm*. Despite some discrepancies in individual rankings, *RP49* and *ATP1A1* were consistently exhibited a higher level of stability than the rest of the candidates projected by all four algorithms (Table 3), suggesting the importance of (1) using a comprehensive analysis to interpret the dataset and (2) adopting the multiple instead of a single normalizer for RT-qPCR analysis.

In recent years, researchers have been more receptive to use multiple reference genes to replace a single normalizer in RT-qPCR analysis^44^. The optimal number of reference genes is typically determined by *geNorm*. In this study, three reference genes for recommended for different developmental stages (*18S, HSP70*, and *28S*), tissues (*28S, 18S*, and *RP49*), temperatures (*18S, 28S* and *GAPDH*), and photoperiods (*18S, 28S* and *HSP90*), while two reference genes were required for the reliable normalization in different sexes (*HSP90* and *RP49*), and *in vivo* RNAi (*RP49* and *ATP1A1*). Our combined results are, in part, consistent with previous studies of other Coccinellidae predatory species (Table 4), especially for ribosome RNAs (rRNAs).

**Table 4:** Recommended reference genes for RT-qPCR Analysis in Coleoptera.

Species	Biotic Conditions			Abiotic Conditions			Others
	Dev. Stage^*^	Tissue	Sex	Temperature	Photoperiod	RNAi	
*Coccinellidae*							
*Harmonia axyridis* (this study)	*18S, HSP70, 28S*	*28S, 18S, Rp49/RpL32*	*HSP90, Rp49/RpL32, HSP70*	*18S, 28S, GAPDH*	*18S, 28S, HSP90*	*Rp49/RpL32, ATP1A1, 28S*	
*Hippodamia convergens*^45^	*28S, EF1A, CypA*	*GAPDH, 28S, CypA*	*GAPDH, CypA, 28S*	*EF1A, 28S, ATP6V1A*	*CypA, GAPDH, ATP6V1A*	*CpyA, Actin, GAPDH*	
*Coleomegilla maculate*^46^	*ATP6V1A, RPS18, EF1A*	NA**	*16S, HSP70, RpS18*	*18S, TUBA, 12S*	NA	*18S, 16S, 12S*	
*Coccinella septempunctata*^47^	*16S, 28S, NADH*	*28S, 16S, 18S*	NA	NA	NA	*ACTB, TUBA, EF1A*	
*Chrysomelidae*							
*Diabrotica virgifera virgifera*^57^	*ACTB, EF1A, RpS9*	*EF1A, GAPDH, TUBB*	NA	NA	NA	*RpS9, EF1A, GAPDH*	*EF1A, GAPDH, TUBB* (Bt)
*Leptinotarsa decemlineata*^58^	*RP18, ARF1, RP4*	*RP18, ARF1, RP4*	NA	NA	NA	NA	*RP18, RP4, ARF1* (Insecticide)
*Galeruca daurica*^59^	*SDHA, Rp49/RpL32, GST*	*SDHA, TUBA, Rp49/RpL32*	*ACTB, TUBA, SDHA*	*SDHA, TUBA, ACTB*	NA	NA	*SDHA, TUBA, GAPDH* (Diapause)
*Cerambycidae*							
*Anoplophora glabripennis*^60^	NA	*Rp49/RpL32, GAPDH, SDF* (Adults)	NA	NA	NA	NA	*GAPDH, UBQ, Rp49/RpL32* (Larvae)
*Tenebrionidae*							
*Tribolium castaneum*^61,62^	NA	NA	NA	NA	NA	NA	*RPS3, RPS18, RPL13a* (Fungus) *RpL13A, RpS3, ACTB* (UV)
*Meloidae*							
*Mylabris cichorii*^63^	NA	NA	*TAF5, UBE3A, RPL22e (Male)*	NA	NA	NA	*UBE3A, RPL22e, TAF5 (Female)*

*Developmental stages.

**Not Applicable. Please note that the abbreviation of gene names may differ among the cited references.

Not surprisingly, rRNAs (e.g., *18S* and *28S*) were consistently stably expressed throughout the vast majority of biotic and abiotic conditions among the four Coccinellidae species, including *H. axyridis*, *Hippodamia convergens*^45^, *Coleomegilla maculate*^46^, and *Coccinella septempunctata*^47^. The over-representation of rRNAs in the total RNA pool (>80%), however, can potentially mask the subtle changes of the target gene expression^48^. Therefore, customized reference gene study is still a prerequisite for standardized RT-qPCR analysis in predatory lady beetles. A large body of works has demonstrated that there are no “universal” reference genes applicable for all cell and tissue types and various experimental conditions^49^. As a major structural protein, *Actin* has been used extensively as the internal control without any validation. In this study, however, *Actin* was one of the least stable candidates under both biotic and abiotic conditions, except the temperature treatment, which is consistent with the other three Coccinellidae species^45–47^.

This study not only provides a standardized procedure for the quantification of gene expression, but also lays a foundation for the genomics and functional genomics dissection of *H. axyridis*, an emerging model in invasion biology^50^.

## Materials and Methods

### Insects

*Harmonia axyridis* was originally collected from the University of Kentucky North Farm (38°07′N, 84°30′W). *Harmonia axyridis* colony was maintained at 23 ± 1 °C, 12 L:12D photoperiod, 50% relative humidity, and provisioned with pea aphids and sugar water for more than two months. Pea aphid clones were a gift from Dr. John Obrycki (University of Kentucky) and maintained on seedlings of fava beans in a glasshouse.

### Experimental conditions

#### Biotic conditions

The developmental stages include eggs (N = 15), four larval instars (N = 5 for each instar, respectively), pupae (N = 1), and adults (one male and one female). Sex of adult beetles was determined by the presence or absence of the male genitalia. Tissues, including head, midgut, and carcass, were dissected from the fourth instar larvae (N = 5).

#### Abiotic conditions

To examine the effects of temperature, third instars were exposed to 10, 22, and 30 °C for 3 hours. For photoperiod, third-instar larvae were treated with a series of light and dark regime of 16 L:8D, 12 L:12D, and 8 L:16D for two days. For *in vivo* RNAi, *H. axyridis ATP6V1A* was the intended molecular target. Specifically, 280 ng of dsRNAs (56 nl, 5 μg/μl), derived from *H. axyridis ATP6V1A* (HA-dsRNA) and a plant gene, *β-glucuronidase* (GUS-dsRNA), were injected into the abdomen of third instars (N = 5). GUS-dsRNA is an exogenous control for the unintended silencing effects, and H_2_O is the vehicle control for the delivery agent of dsRNAs. Samples were collected on day-3 for RT-qPCR analysis.

### Total RNA extraction and reverse transcription

Total RNA was extracted separately from each developmental stage, including eggs (N = 15), pupa (N = 1), and adult (N = 1) for each sex. For other experiments involving larvae, five individuals were pooled as one sample. Each experiment was repeated three times independently. Samples were preserved in 1.5 ml centrifuge tubes and snap frozen immediately in liquid nitrogen before storage at −80 °C. Total RNA was extracted using TRIzol^®^ (Invitrogen, Carlsbad, CA) following the manufacturer’s instructions. Each sample of 2.0 μg RNA was reverse transcribed with random primers using the M-MLV reverse transcription kit (Invitrogen, Carlsbad, CA) according to the manufacturer’s recommendations.

### Primer design and cloning of candidate reference genes

Primers for *18S, 28S, ATP1A1, HSP70, HSP90*, and *RP49* (Table 1) were designed based on their respective sequences from NCBI (http://www.ncbi.nlm.nih.gov/). Degenerate primers for *ACTB*, *GAPDH, ATP6V1A* were designed using CODEHOP (http://blocks.fhcrc.org/codehop.html). PCR amplifications were performed in 50 μl reactions containing 10 μl 5 × PCR Buffer (Mg^2+^ Plus), 1 μl dNTP mix (10 mM of each nucleotide), 5 μl of each primer (10 μM each), 0.25 μl of Go Taq (5 u/μl) (Promega, Madison, WI) and 25 ng first-strand cDNA. The PCR parameters were as follows: one cycle of 94 °C for 3 min; 35 cycles of 94 °C for 30s, 55 °C for 1 min and 72 °C for 1 min; a final cycle of 72 °C for 10 min. PCR products were purified and cloned into the pCR™4-TOPO® vector (Invitrogen, Carlsbad, CA) for sequencing confirmation. The primers for the target gene, *TPS*, were obtained from a previous work^51^.

### Quantitative real-time PCR (RT-qPCR)

Gene-specific primers (Table 1) were used in PCR reactions (20 μl) containing 7.0 μl water, 10.0 μl 2 × SYBR Green MasterMix (BioRad, Hercules, CA), 1.0 μl each specific primer (10 μM), and 10 ng first-strand cDNA. The RT-qPCR program included an initial denaturation for 3 min at 95 °C followed by 40 cycles of denaturation at 95 °C for 10 s, annealing for 30 s at 55 °C, and extension for 30 s at 72 °C. For melting curve analysis, a dissociation step cycle (55 °C for 10 s, and then 0.5 °C for 10 s until 95 °C) was added. Three technical replicates were analyzed for each biological replicate.

Reactions were performed in a MyiQ Single Color Real-Time PCR Detection System (BioRad). The existence of one peak in melting curve analysis was used to confirm gene-specific amplification and to rule out non-specific amplification and primer-dimer generation. The RT-qPCR was determined for each gene using slope analysis with a linear regression model. Relative standard curves for the transcripts were generated with a serial dilution of cDNA. The corresponding RT-qPCR efficiencies (E) was calculated according to the equation:

$${\rm{E}}=({{\rm{10}}}^{[-{\rm{1}}/{\rm{slope}}]}-{\rm{1}})\times 100 \% {\rm{.}}$$

### Stability of gene expression

The stability of the nine candidate reference genes were evaluated using *RefFinder* (http://www.leonxie.com/referencegene.php), a web-based analysis tool which integrates all four major computational programs, including *geNorm*^31^, *NormFinder*^52^, *BestKeeper*^53^, and the comparative ΔCt method^54^. *geNorm* calculates an expression stability value (M) for each gene and a pair-wise comparison. *NormFinder* ranks the set of candidate genes based on their expression stability in the given sample set. *BestKeeper* considers the Ct values of all candidate reference genes, to calculate standard deviation and coefficient of variation. ΔCt approach directly compares relative expression of ‘pairs of genes’ within each sample. Then, *RefFinder* assigned an appropriate weight of the four methods to an individual gene and calculated the geometric mean of their weights for the overall final ranking.

### Validation of selected reference genes

*Trehalose-6-phosphate synthase* (*TPS*), the intermediate of trehalose, is a key component in insect energy metabolism and resilience^25,51,55^. The stability of candidate reference genes was evaluated using *TPS* as the target gene. *TPS* expression levels under different temperature treatments were calculated based on selected sets of candidate reference genes. Two separate normalization factors (NFs) have been computed based on (1) the geometric mean of the genes with the lowest *Geomean* values (as determined by *RefFinder*), and (2) a single normalizer with the lowest or highest *Geomean* value. Relative expression of *TPS* in different samples was calculated using the 2^−ΔΔCt^ method^56^.
